# Supplementary figures and images for: Transcriptome of the parasitic flatworm Schistosoma mansoni during intra-mammalian development
Source: PLoS Negl Trop Dis. 2020 May 6;14(5):e0007743. doi: 10.1371/journal.pntd.0007743 (PMC7263636; doi:10.1371/journal.pntd.0007743)

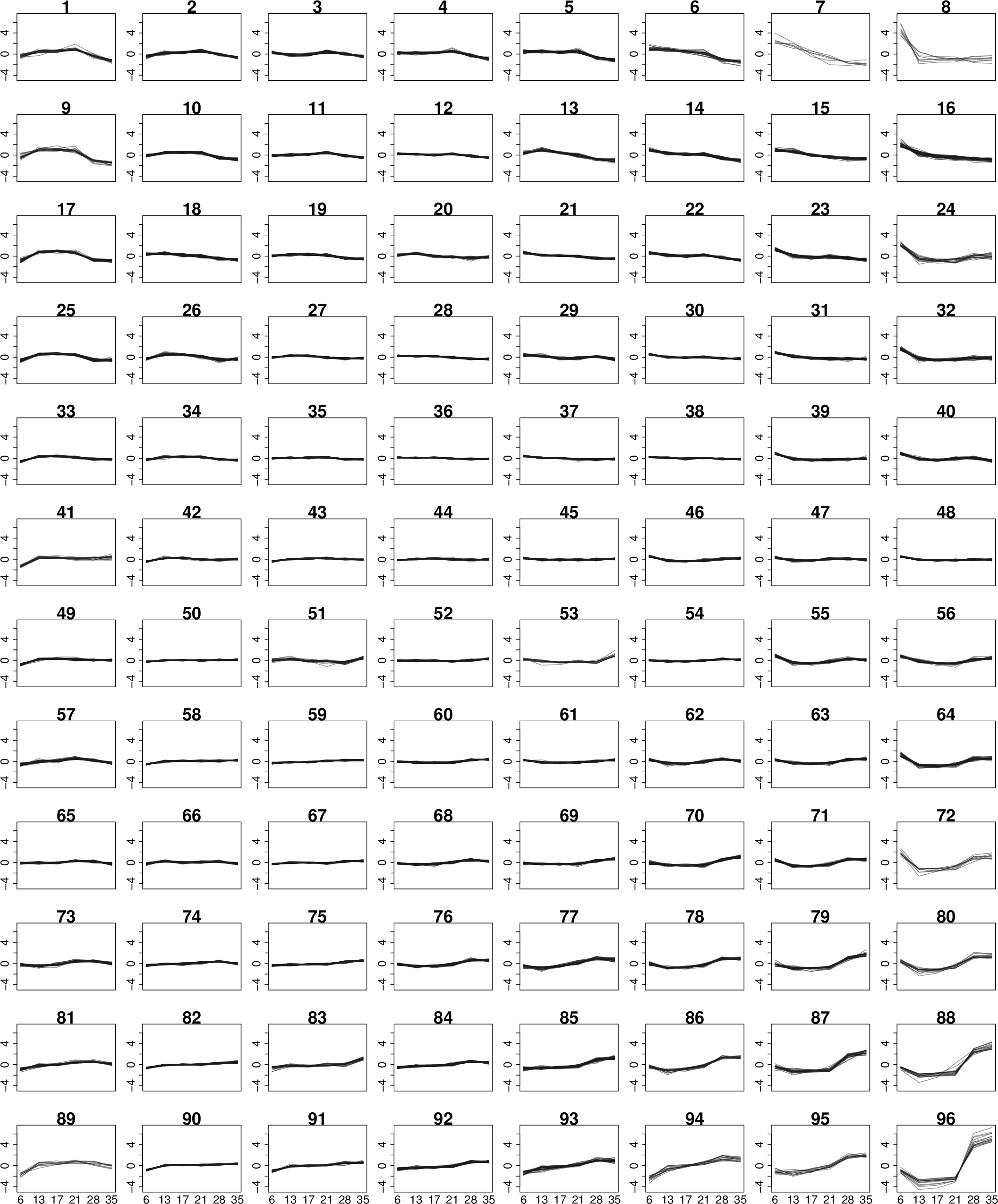

Supplement: S1 Fig — Expression profile of genes differentially expressed in at least one time point clustered into 96 groups. The clustering was done on mean-normalised regularized log transformation (rlog- transformed) of raw read counts. X-axes represent six time points from this dataset; y-axes represent the mean-normalised rlog-transformed. Unlike Fig 4, this supplementary figure shows clusters with a fixed range for the y-axis across all clusters in order to visualise clusters with the largest changes over time. (TIF) [file pntd.0007743.s001.tif]

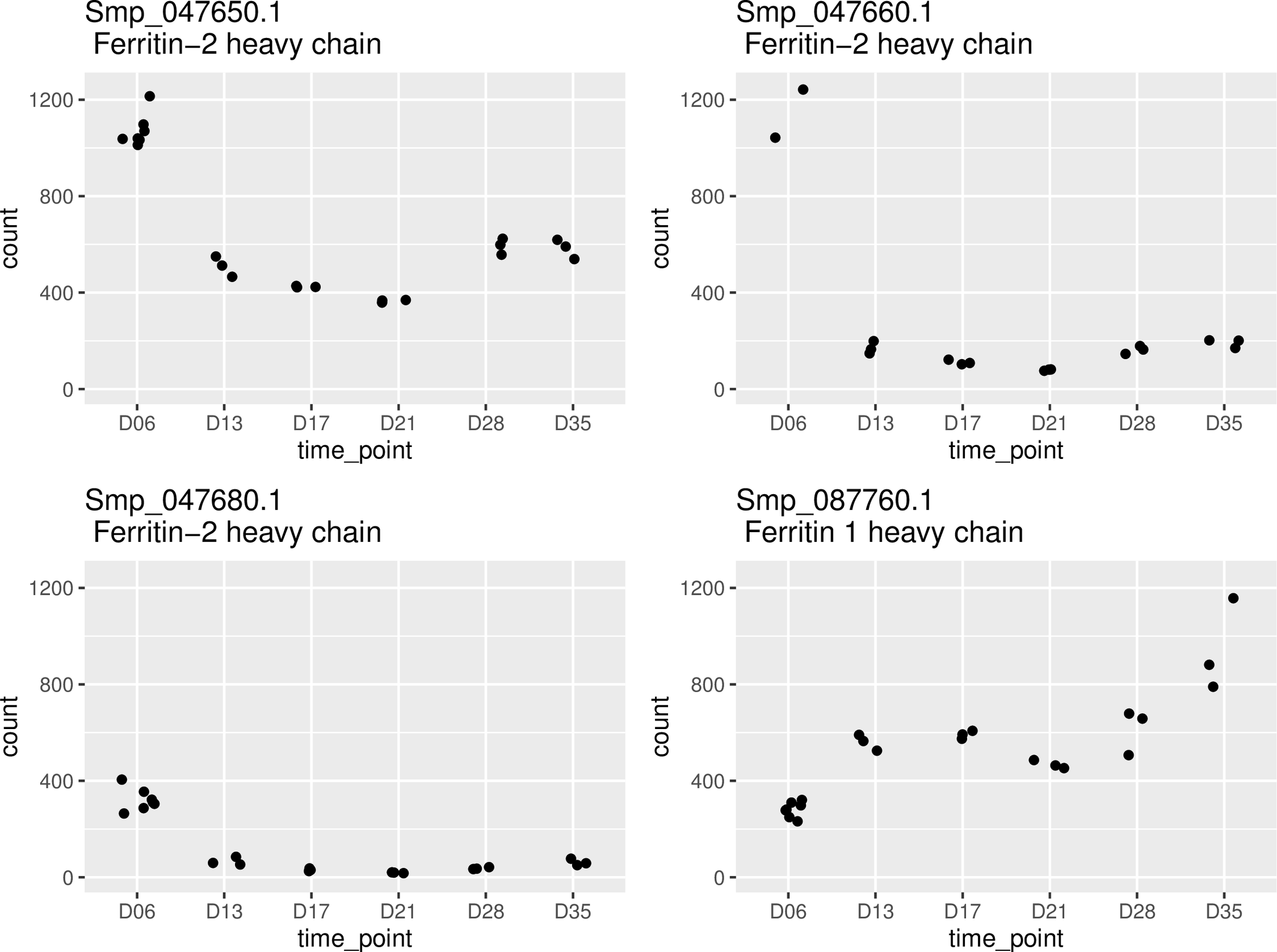

Supplement: S2 Fig — Each dot represents one replicate from each of the time points. Y-axis represents normalised counts from DESeq2. (TIF) [file pntd.0007743.s002.tif]

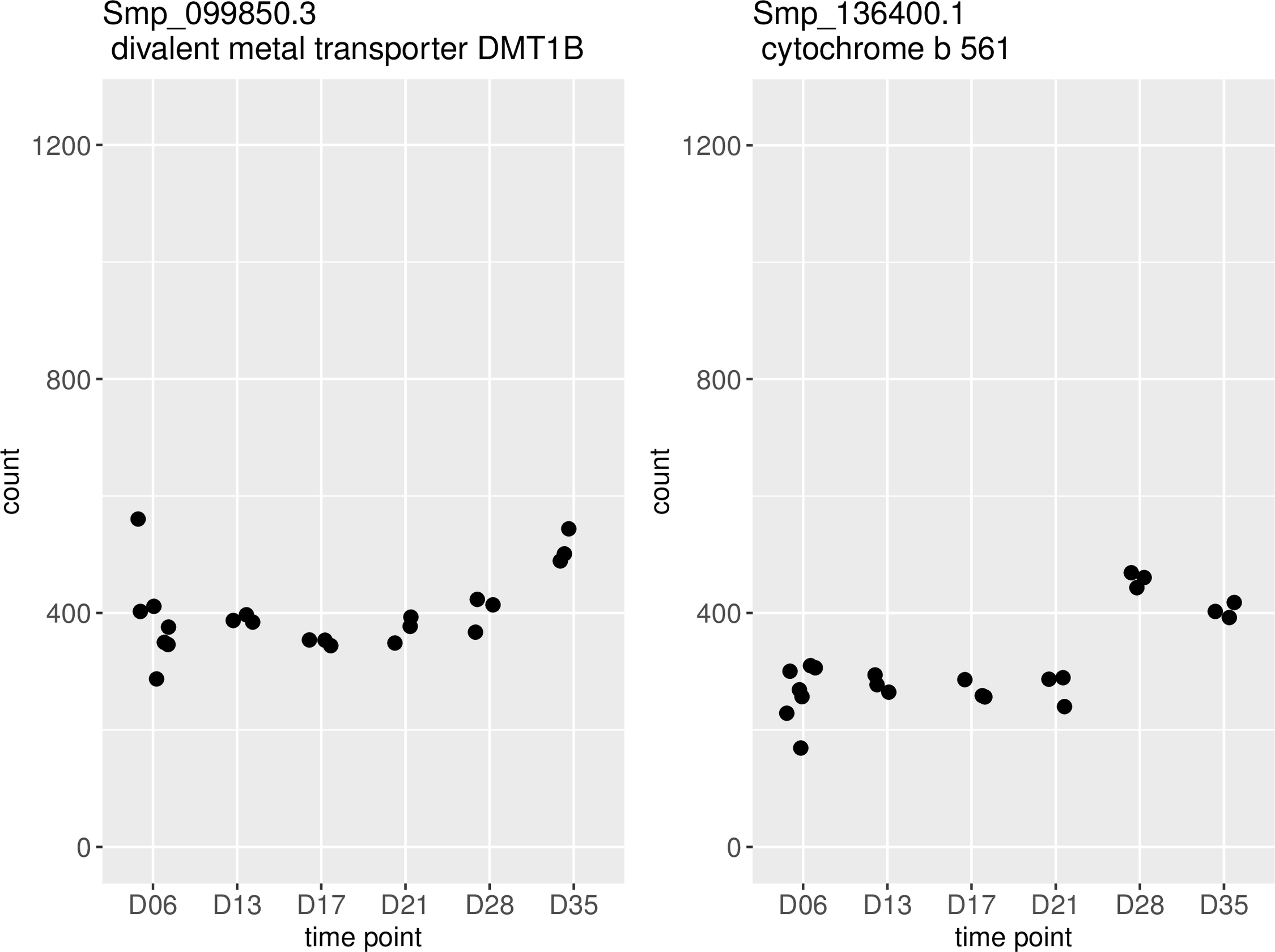

Supplement: S3 Fig — Each dot represents one replicate from each of the time points. Y-axis represents normalised counts from DESeq2. (TIF) [file pntd.0007743.s003.tif]

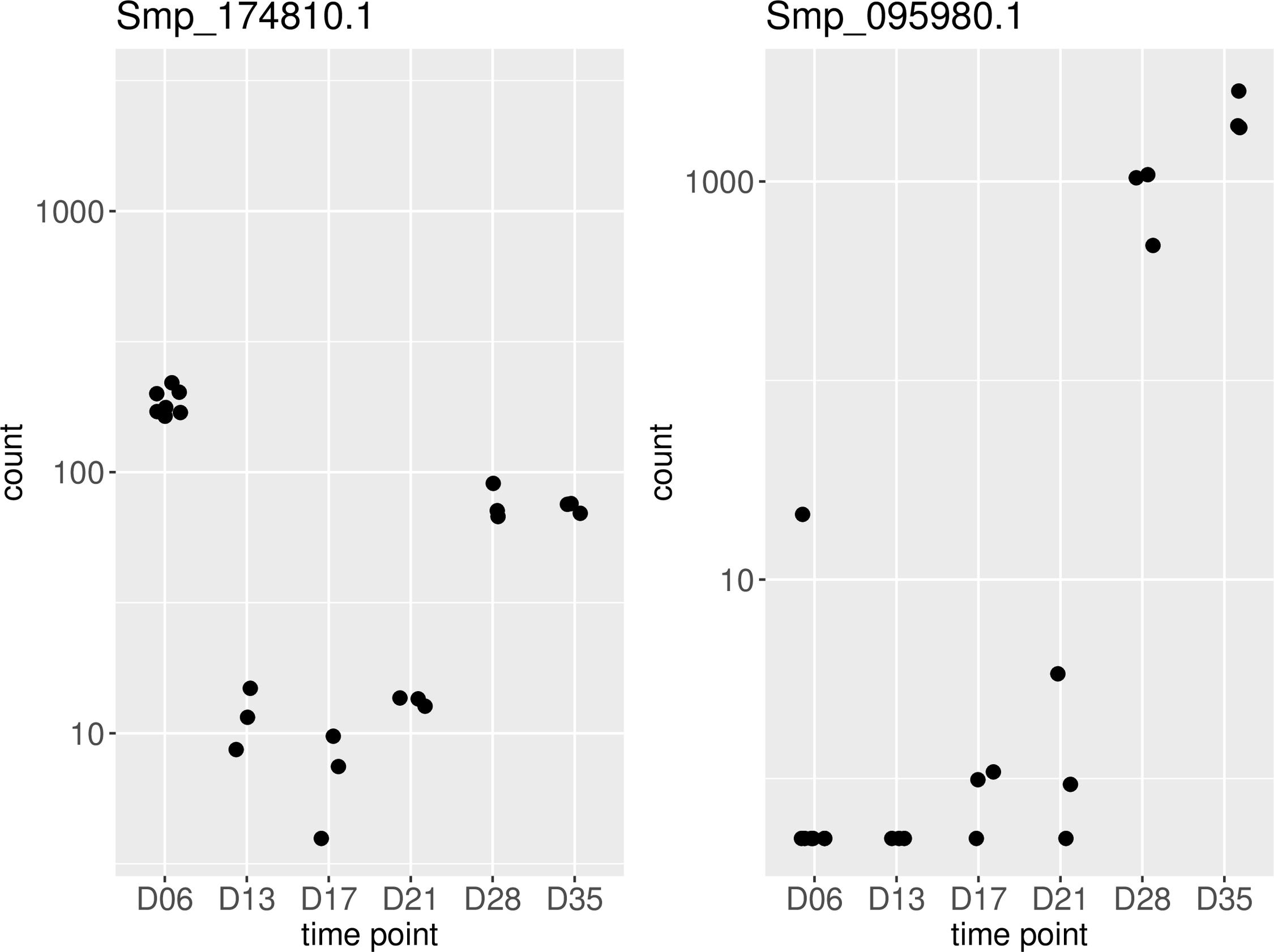

Supplement: S4 Fig — Expression of Smp_174810 and Smp_095980 with y axes on log scale. Each dot represents one replicate from each of the time points. Y-axis represents normalised counts from DESeq2. Smp_095980 was identified as differentially expressed (S2 Table and S4 Fig), but this figure shows that its expression in the lung stage was high in only one out of seven replicates. (TIF) [file pntd.0007743.s004.tif]

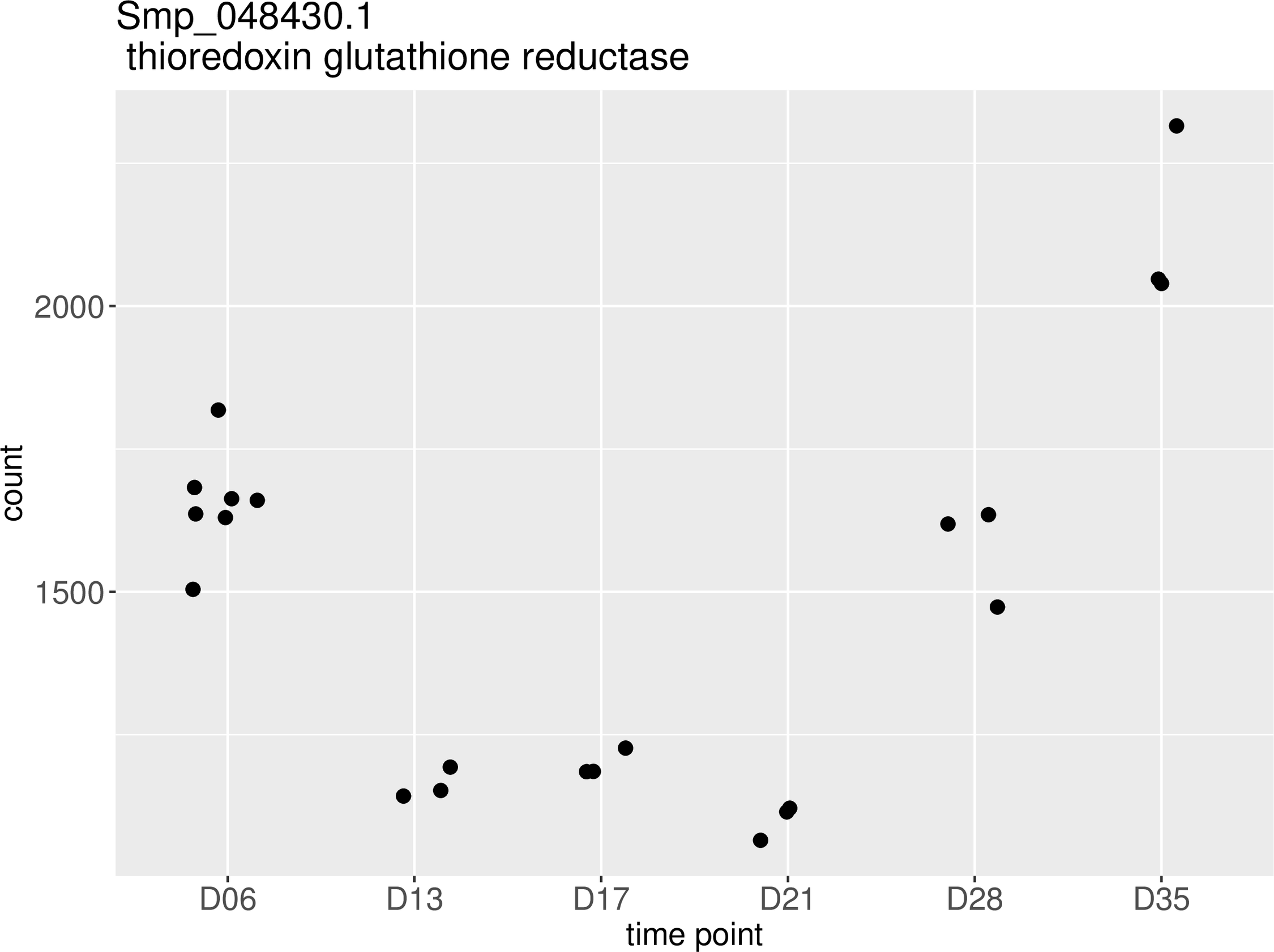

Supplement: S5 Fig — Each dot represents one replicate from each of the time points. Y-axis represents normalised counts from DESeq2. Log2FC between D13/D06 is -0.52, adjusted p-value for differential expression between D13/D06 is 4.10e-21. (TIF) [file pntd.0007743.s005.tif]

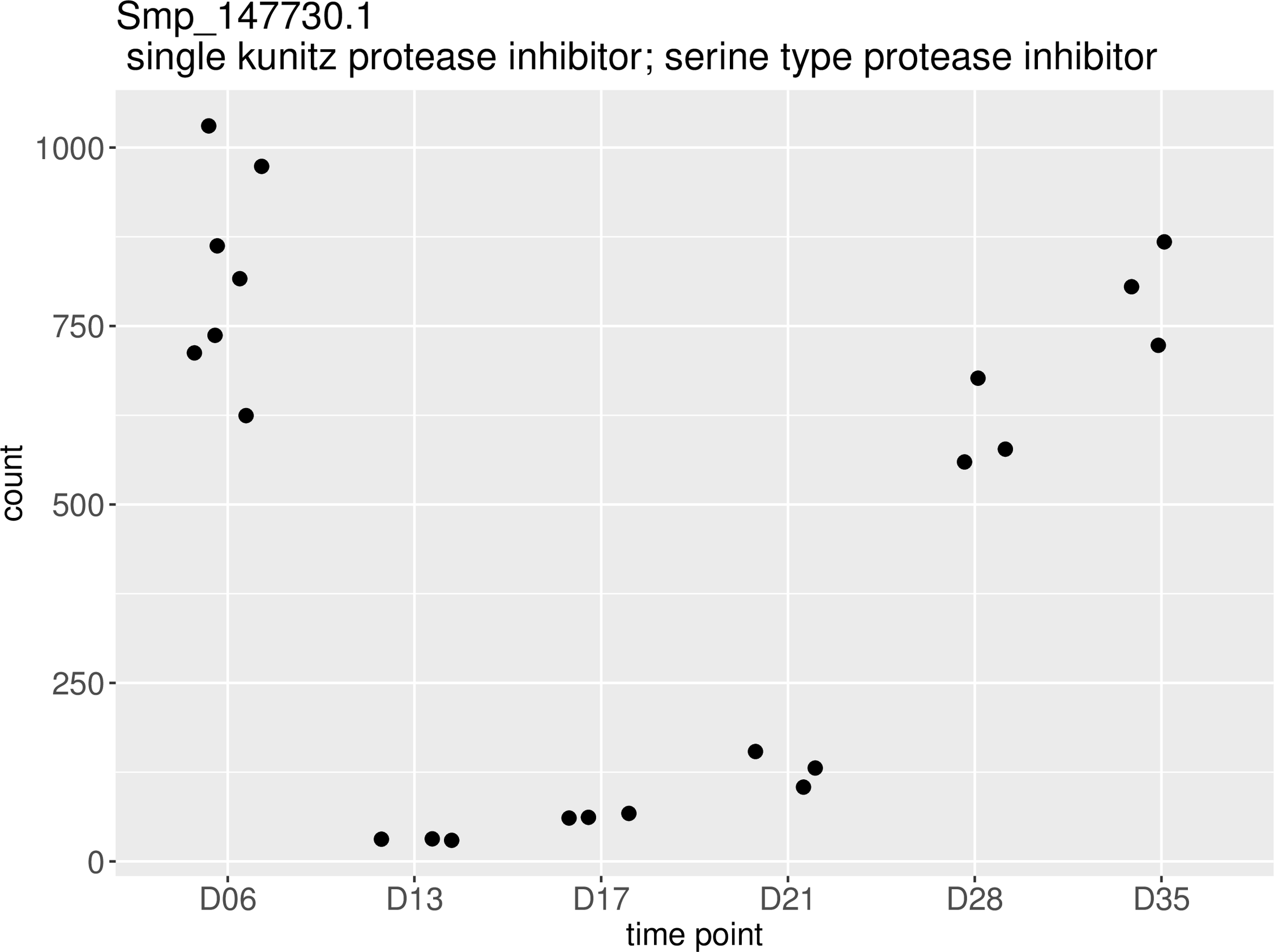

Supplement: S6 Fig — Each dot represents one replicate from each of the time points. Y-axis represents normalised counts from DESeq2. (TIF) [file pntd.0007743.s006.tif]

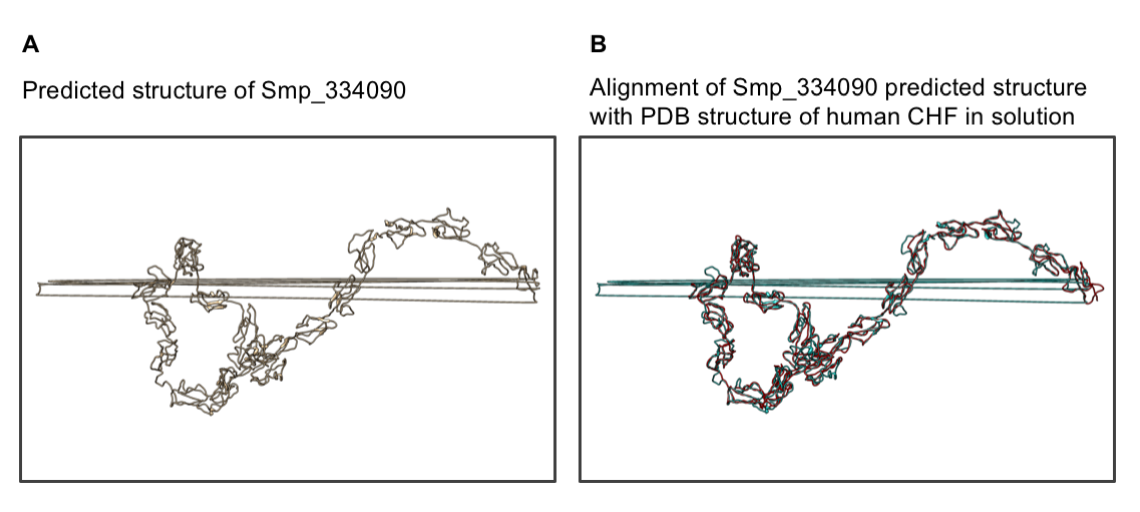

Supplement: S7 Fig — A) Predicted 3D structure of Smp_334090 (resulting from a merge of Smp_182770 and Smp_038730 in the most recent version of the S. mansoni genome) by I-TASSER based on the amino acid sequence. B) Alignment between the predicted structure (blue) and 3D structure of human CFH (in 137 mM NaCl buffer) obtained from PDB (PDB identifier: 3GAV) (red). (TIF) [file pntd.0007743.s007.tif]
