# Supplementary material for: Transcriptome of the parasitic flatworm Schistosoma mansoni during intra-mammalian development
Source: PLoS Negl Trop Dis. 2020 May 6;14(5):e0007743. doi: 10.1371/journal.pntd.0007743 (PMC7263636; doi:10.1371/journal.pntd.0007743)
Supplement: S1 File — Total number of reads and % mapped to S. mansoni genome for each sample. (DOCX) [file pntd.0007743.s022.docx]

**S1 File. RNA-seq details**

| Sample (named as stage_replicate) | Total number of reads | % mapped to *S. mansoni* genome | Average % mapped to  *S. mansoni* genome |
| --- | --- | --- | --- |
| D06_1 | 19,318,784 | 49.90 | 40.21 |
| D06_2 | 23,925,410 | 35.93 |  |
| D06_3 | 23,574,302 | 39.25 |  |
| D06_4 | 22,251,402 | 39.36 |  |
| D06_5 | 21,602,356 | 40.85 |  |
| D06_6 | 21,931,338 | 37.43 |  |
| D06_7 | 17,379,984 | 38.72 |  |
| D13_1 | 22,506,450 | 90.03 | 88.01 |
| D13_2 | 31,002,978 | 86.39 |  |
| D13_3 | 22,999,580 | 87.61 |  |
| D17_1 | 27,001,864 | 90.45 | 87.76 |
| D17_2 | 13,977,452 | 87.54 |  |
| D17_3 | 17,331,172 | 85.29 |  |
| D21_1 | 14,092,426 | 93.51 | 88.70 |
| D21_2 | 17,310,410 | 92.10 |  |
| D21_3 | 17,928,990 | 80.48 |  |
| D28_1 | 21,449,746 | 84.17 | 88.86 |
| D28_2 | 18,490,898 | 90.58 |  |
| D28_3 | 17,843,522 | 91.82 |  |
| D35_1 | 14,515,718 | 91.36 | 88.32 |
| D35_2 | 19,775,508 | 85.99 |  |
| D35_3 | 14,843,052 | 87.60 |  |

Compared to data from other time points, RNA-seq data from the day-6 (lung stage) worms contained fewer reads that mapped to *S. mansoni* genome. A large number of day-6 data mapped to the rodent genomes. This was expected given the collection protocol that involved incubating chopped lung to recover the worms.
