# Supplementary material for: Transcriptome of the parasitic flatworm Schistosoma mansoni during intra-mammalian development
Source: PLoS Negl Trop Dis. 2020 May 6;14(5):e0007743. doi: 10.1371/journal.pntd.0007743 (PMC7263636; doi:10.1371/journal.pntd.0007743)
Supplement: Attachment — Submitted filename: Author response Wangwiwatsin et al.docx [file pntd.0007743.s025.docx]

We are grateful to the reviewers for their constructive comments. All three expressed enthusiasm but also raised specific issues that are addressed point-by-point below.

Reviewer #2: The objectives of the study are clear and study design is appropriate as

presented, but I am concerned that previous knowledge from studies of additional life cycle stages are not discussed.

We agree that a considerable corpus of literature exists based on a combination of studies across different developmental stages. Indeed, we relied heavily upon the literature (and cross-referenced protein databases) to identify key genes and infer functional implications. We have re-written the introduction to better emphasise the prior work highlighting our aim of bringing uniformity to comparisons across a number of clinically important stages. Given our focus, results that stood out, and led to discussion points, were largely related to mammalian stages. Qualitative comparisons with other studies were of course possible and we endeavoured to include many (but without exhaustively describing every gene). We have released an attempt to combine data from across studies into a full meta-analysis that uses the TPM method of normalisation that is used in many other studies/ resources. The study is indeed available via the BioRxiv (<https://doi.org/10.1101/308189>) and many researchers have found it a useful starting point for data exploration but the approach is fraught with normalisation challenges, particularly in samples with heterogeneous expression and variable numbers of replicates (as reviewed in PMID:26813401). At present, we are convinced that comparing samples from within one study is really the only reliable approach.

Minor comments:

1.	(Line 77) Previous studies on intra-mammalian development of *S. mansoni* were mentioned and it would have been helpful for the general audience to include statements on the major findings/summaries from those in vitro/in vivo studies.

The introduction has been re-written and now includes better referencing to the literature, summarising major contributions from *in vitro* and *in vivo* studies in this area.

2.	Authors should include details of the raw RNA-seq data including number of reads obtained and % of mapping.

The suggested details have been now added to supplementary file S1

Reviewer #2: The conclusions are supported but the limitations could be better addressed.

Following the reviewer’s suggestion we have inserted the following paragraph into the discussion.

“Certain limitations need to be borne in mind when interpreting the data from this study. First, it has been shown that gene expression can change with the types of hosts (95,96) and, therefore, this limits extrapolation of findings from mouse host to humans. In *S. japonicum*, microarray data of adult worms collected from two different mouse strains, rabbits and water buffaloes (natural host) displayed highly correlated expression profiles across different host types. Nevertheless, over 400 genes were identified as differentially expressed, particularly including those likely to be involved in signal transduction and metabolism (95). Second, our transcriptome data were produced by ‘bulk’ RNA-seq from pooled mixed-sex worms. In bulk RNA-seq strong signals from rarer cells types can be averaged out, and gene expression changes can therefore be missed if, for instance, a gene is up-regulated in one cell type but down-regulated in another. Single-cell transcriptomic analyses promise to overcome this limitation (97). Using mixed-sex worms in our study was another possible confounder for genes, such as ferritin-1, where there is differential expression between male and female worms. However, the majority of our analyses focused on earlier stages where the sexes show little or no differentiation. Despite these various caveats that are common to many bulk analyses, the good agreement with key known events during parasite development provides confidence that the major transcriptional signatures we have detected reflect major changes within the parasite.”

**Reviewer #1:**

1. Line 262. Upregulation of molecules associated with neuronal function. One very interesting analysis of Fasciola development in its mammal host by MV Sukhdeo of Rutgers University suggested that trematodes followed fixed actions patterns of behaviour, that is, the parasites displayed certain behaviours that facilitated their development to a certain point. Having reached that point, a whole new set of behaviours were initiated. The lung stage of Schistosoma seems critical in development and host-parasite interaction and some major morphological changes occur in the parasites in the time they are present in the lungs. So, just as a thought exercise, I wonder if it is worthwhile adding that the neuronal activity is also associated with a transition to a whole new phase of development?

We thank the reviewer for this suggestion. It is an interesting point and has been incorporated into the Discussion as follows:

“...In some parasitic trematodes, fixed behavioural patterns have been associated with specific developmental stages involved in processes such as migration, tissue-tropism, and adhesion (76,77). Given that the lung stage undergoes major morphological changes, an increase in neuronal activities indicated by our data may reflect distinct behaviours associated with the new phase of development, as well as responses to a new environment within the lung capillaries. Neuronal signalling could involve both the sensing of stimuli and motor responses being one of the biological processes disturbed in irradiated schistosomes (78) that exit lung capillaries into the alveoli, eventually dying during the lung migration (20,74,79).”

2. I was happy to see reference to iron metabolism and the putative iron transporters and related genes. The findings of ferritin are interesting. Work on S mansoni (Schüssler et al Mol Reprod Dev. 1995 Jul;41(3):325-30) and S japonicum (Jones 2007;39(9):1646-58) demonstrate that one form of ferritin (called yolk ferritin) is associated with vitelline cells. Iron was proposed to be either stored in vitelline cells as part of the requirements for development in the snail (Schüssler) or to stabilise the egg shells (Jones). One would expect or hope to see one ferritin shoot up in its transcriptomic profiles in older worms. Is there any evidence of this occurring. Given the statement above, does the drop in ferritin levels at day 13 seem somewhat strange?

The drop in ferritin expression did seem surprising as iron is required for development and reproduction, but in fact the isoforms that dropped after the lung stage were annotated as ferritin-2. As shown in Figure S2, there was still some expression of ferritin during the developmental and adult stages. As mentioned in our discussion, the higher expression of genes encoding ferritins in the lung stage may reflect roles of ferritins for iron sequestration and immune response modulation. Regarding the yolk ferritin (ferritin-1), this gene did show increased expression over the time course and did shoot the highest in egg-laying adult stage. We have made a clearer distinction between ferritin-1 and ferritin-2 in the results, added the expression plot of ferritin-1 gene (Smp_087760) to Figure S2, and including the references suggested by the reviewer.

The text previously stated:

“...the *ferritin*-related genes (Smp_047650, Smp_047660, Smp_047680) displayed fold changes in the range 2.1–7.2 (S2 and S3 Tables). These three *ferritin* genes, although down-regulated in day-13 compared to lung stage schistosomula, were still expressed at a low level during day 13 when iron is required for growth and development (S2 Fig).”

The modified version states:

“...the *ferritin*-2 genes (Smp_047650, Smp_047660, Smp_047680; somal ferritin) displayed fold changes in the range 2.1–7.2 (S2 and S3 Tables). Although down-regulated in day-13 compared to lung stage schistosomula, these three *ferritin* genes were still expressed at a time when iron is required for growth and development (S2 Fig). Another ferritin isoform, ferritin-1 (Smp_087760; yolk ferritin), is associated with vitelline cells (51,52) and in the present study showed increasing expression as the parasite developed into adult stage (S2 Fig). However, sex-biased expression of ferritin-1 and 2 has previously been reported (51,52) and in our mixed-sex samples, we cannot rule out the possibility that our later collection time points contained a greater proportion of female worms.”

3. I may have missed it, but do the authors have a way of determining the numbers of males and females in each host at different stage? Is the gender balance the same in all mice? Implicit in this question is the concept that males and females will, or be expected to, have distinct transcriptional profiles at all stages. Would a difference in gender balance between mice or between stages skew the transcriptomes? Thus, is there a way of, using either male-only or female-only genes as markers of the numbers of respective gender balance?

It is not possible, to our knowledge, to determine the numbers of males and females in each host from transcriptomic data for two main reasons. First, it is believed that the molecular differentiation for male (ZZ) and female (ZW) *S. mansoni* relies on female-specific markers that are not present in males.

The ratio between male and females within each host is now mentioned in relation to interpreting the ferritin results (see above) and is mentioned in the discussion as a specific caveat of this work.

**Reviewer #2:** The research presented here in the manuscript of Wangwiwatsin et al, is experimentally sound and examines difficult to study in vivo lifecycle stages with the power of and quantitative advantages of RNA-seq. Of the work that is presented I have almost no concerns and several interesting findings, especially the CHF and other immune-related findings. My concerns and request for major revision are focused on what is not presented. In particular:

1) The source of the S. mansoni parasites used is not presented.

The information on *S. mansoni* strain has been added to the Materials and Methods under the “Mouse infection” section. The parasites were from the life cycle maintained by Mike J. Doenhoff at the University of Nottingham and this information is part of the Ethics Statement.

2) There is no discussion of potential parasite gene expression differences in a mouse vs human host.

We thank the reviewer for raising this point. Please see earlier comment, a section on caveats/limitations has now been added to the discussion.

3) The references to previous RNA-seq analysis of Schistosoma gene expression including S. mansoni is quite lacking, only much older literature is cited. In fact, this manuscript fails to cite a previous Berriman bioRxiv analysis of existing RNA-seq data

Please see the earlier comment. We have now re-written the introduction, including much better referencing to the published literature. The reviewer mentions an unpublished study in the BioRxiv by Lu et al. The latter study attempted a meta-analysis across life cycle stages from numerous studies. However normalisation across study designs is fraught with problems. In our unpublished BioRxiv paper we describe a resource where a relatively crude TPM normalisation method is used. The approach has been widely used by others but is difficult to validate. Our introduction therefore only reflects the published literature.

4) Despite the fact that the S. mansoni genome is now on version 8, with version 7 publicly released by the Wellcome Trust for more than a year in WormBase Parasite, the much older assembly version 5 was sued. This seems odd. In fact, the MS has to go to this newer version to highlight a gene of interest that is mis-assembled in version 5.

Version 7 of the S. mansoni genome has not yet been published and a stable version 8 is not available. However, we are sympathetic with the reviewer in that our policy of early data release pre-publication has in this case created an anomaly. As with many large scale omics projects, data analysis has to be based on a static snapshot of the data and by the time the analysis has been completed the data have often been migrated to a new version.

Between v5 and v7 (note, v6 was released only as an interim step to facilitate some external genome-scale projects by others), there have been a lot of changes to the assembly and these will be described in a future manuscript. The analysis in the present work is based on protein coding sequences and these have changed to a lesser degree between versions. In the majority of cases, predicted gene structures and identifiers have been preserved between versions. There are exceptions, and this is why key results were manually reconciled against v7 to ensure that our conclusions were solid.

The major versioning difference affected some genes that displayed border-line differential expression and this in turn affected the enrichment scores of some GO terms. Apart from these differences, the results of the v7 analyses yielded consistent key messages with those of v5. For instance, the expression of the CFH-like genes followed the same expression profile.

4) Most significantly, this manuscript only focuses on the data they have generated and fail to include gene expression insights from the rest the lifecycle. It is impossible to declare, as in line 321 "Lung-stage specific" when all stages are not considered. Given that the RNA-seq data for other stages exists and findings from these studies and microarrays exist these new data should be analyzed in that context.

We agree with the reviewer that our language here was quite loose. We thank the reviewer for pointing out the misleading term and have changed **“lung stage-specific”** to **“Genes with high expression in the lung stage compared to other intra-mammalian stages”.**

As responded above, analysis of transcriptomes from the all life cycle stages is beyond the scope of this current study which focuses on intra-mammalian stages. More importantly, cross-analysing data that come from different platforms, host strain, and different sample handling methods, have significant limitations.

5) Minor comment line 379 "appeared to [be] upregulated" what is the uncertainty?

We thank the reviewer for pointing this out. Uncertainty was unintentionally emphasized. The text has been changed to **“multiple genes related to signaling processes were up-regulated”.**

In addition to changes to address reviewer comments, the following text has been changed to better emphasize that the data are publicly available:

“In a subsequent version of the *S. mansoni* genome (unpublished), the two genes have been merged to become Smp_334090 (data available on WormbaseParasite.org).”

Now reads:

“In a subsequent unpublished version of the *S. mansoni* genome (available from parasite.wormbase.org), the two genes have been merged to become Smp_334090.”
